# An Inorganic–Organic Hybrid Framework Composed of Polyoxotungstate and Long-Chained Bolaamphiphile

**DOI:** 10.3390/ijms24032824

**Published:** 2023-02-01

**Authors:** Haruka Ikuma, Shunsuke Aoki, Kai Kawahara, Seiji Ono, Hironori Iwamatsu, Jun Kobayashi, Yoshiki Kiyota, Yosuke Okamura, Masashi Higuchi, Takeru Ito

**Affiliations:** 1 Department of Chemistry, School of Science, Tokai University, Kanagawa 259-1292, Japan; 2 Department of Applied Chemistry, School of Engineering, Tokai University, Kanagawa 259-1292, Japan

**Keywords:** polyoxometalate, inorganic–organic hybrid, single crystal, surfactant, bolaamphiphile

## Abstract

Surfactants are functional molecules utilized in various situations. The self-assembling property of surfactants enables several molecular arrangements that can be employed to build up nanometer-sized architectures. This is beneficial in the construction of functional inorganic–organic hybrids holding the merits of both inorganic and organic components. Among several surfactants, bolaamphiphile surfactants with two hydrophilic heads are effective, as they have multiple connecting or coordinating sites in one molecule. Here, a functional polyoxotungstate inorganic anion was successfully hybridized with a bolaamphiphile to form single crystals with anisotropic one-dimensional alignment of polyoxotungstate. Keggin-type metatungstate ([H_2_W_12_O_40_]^6−^, H_2_W_12_) was employed as an inorganic anion, and 1,12-dodecamethylenediammonium (C_12_N_2_) derived from 1,12-dodecanediamine was combined as an organic counterpart. A simple and general ion-exchange reaction provided a hybrid crystal consisting of H_2_W_12_ and C_12_N_2_ (C_12_N_2_-H_2_W_12_). Single crystal X-ray structure analyses revealed a characteristic honeycomb structure in the C_12_N_2_-H_2_W_12_ hybrid crystal, which is possibly effective for the emergence of conductivity due to the dissociative protons of C_12_N_2_.

## 1. Introduction

Surfactants are functional molecules utilized in various situations by humans [1,2,3]. Surfactants form several self-assembled structures, such as vesicle, rod, or lamellar structures. Washing function is known to be derived from the formation of surfactant vesicles in solution. These self-assembled structures can be transferred into a solid state to obtain well-ordered nanometer-sized architectures [4]. Utilizing charged surfactants enables the buildup of architectures with charged inorganic species; the optimized combination of the charged species leads to functional inorganic–organic hybrid materials.

Bolaamphiphile surfactants have two hydrophilic heads in one molecule, and are effective components for the preparation of hybrid materials with inorganic species due to their multiple connection or coordination sites [5,6,7]. Inorganic polyoxometalate (POM) anions are promising candidates as an inorganic counterpart owing to their characteristic redox functions [8,9,10,11]. Inorganic–organic POM-surfactant hybrid materials [12,13,14,15,16,17,18] and crystals [19,20,21,22,23,24,25,26,27] have been extensively investigated; however, bolaamphiphile surfactants have rarely been hybridized with POM anions [28,29,30,31,32,33]. Additionally, long bolaamphiphiles with methylene groups ≥7 have rarely been hybridized with POMs; hybridization has been limited to POMs consisting of vanadium [34] and molybdenum [35,36,37,38].

**Figure 1:**
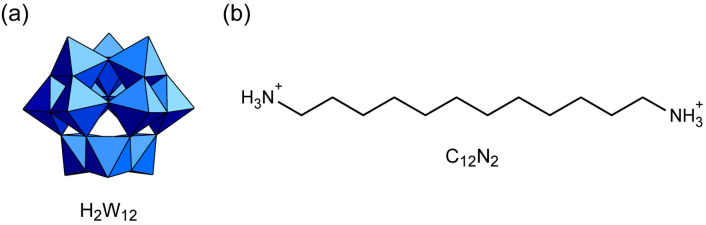
Molecular structure of utilized components: (**a**) Keggin-type metatungstate anion, [H_2_W_12_O_40_]^6−^ (H_2_W_12_); and (**b**) Dodecamethylenediammonium, [H_3_N(CH_2_)_12_NH_3_]^2+^ (C_12_N_2_).

Here, we report an inorganic–organic hybrid crystal consisting of polyoxotungstate (tungsten POM) and a long bolaamphiphile cation with 12 methylene groups. Keggin-type metatungstate anion ([H_2_W_12_O_40_]^6−^, H_2_W_12_, Figure 1a) and dodecamethylenediammonium ([H_3_N(CH_2_)_12_NH_3_]^2+^, C_12_N_2_, Figure 1b) derived from 1,12-dodecanediamine were employed as inorganic and organic motifs, respectively. A hybrid crystal of C_12_N_2_-H_2_W_12_, the first example of tungsten POM with a long bolaamphiphile obtained as single crystals, was synthesized using a simple and general ion-exchange reaction. C_12_N_2_-H_2_W_12_ possessed a characteristic one-dimensional architecture derived from the honeycomb arrangement of the H_2_W_12_ anions. The conductivity of C_12_N_2_-H_2_W_12_ was investigated under low- and high-humidified conditions near room temperature.

## 2. Results

### 2.1. Syntheses of C_12_N_2_-H_2_W_12_ Hybrid Crystal

**Scheme 1:**
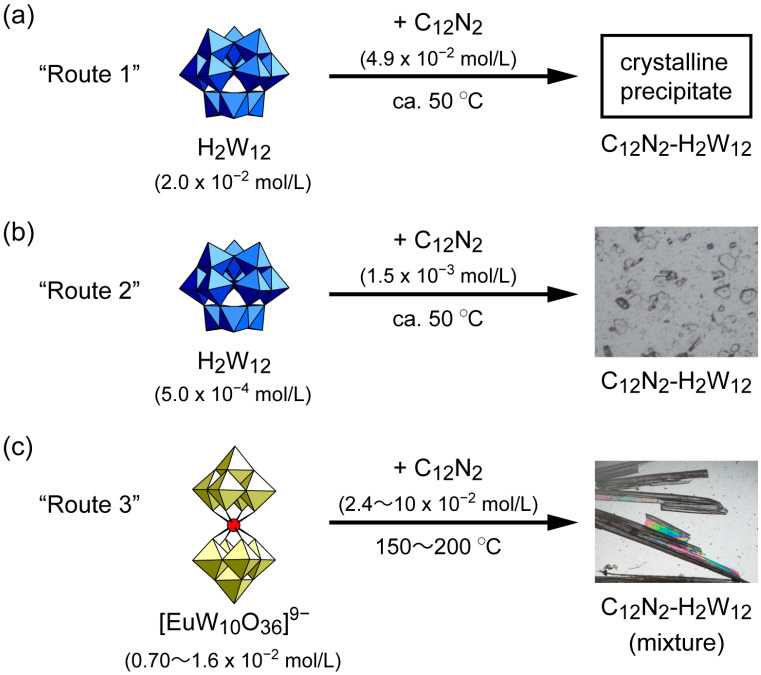
Schematic representations of synthetic procedures for C_12_N_2_-H_2_W_12_ hybrid crystals: (**a**) “Route 1”, an ion-exchange reaction using concentrated solutions; (**b**) “Route 2”, an ion-exchange reaction using diluted solutions; and (**c**) “Route 3”, a hydrothermal reaction using decatungstoeuropate anion as a starting material.

Hybrid crystals of C_12_N_2_-H_2_W_12_ were synthesized using three different procedures, depicted in Scheme 1. The “Route 1” method (Scheme 1a) was an ion-exchange reaction (utilizing a concentrated solution of sodium salt) of H_2_W_12_ anions (Na-H_2_W_12_) and C_12_N_2_ cations derived from 1,12-dodecanediamine, which yielded ~90% colorless crystalline precipitate of C_12_N_2_-H_2_W_12_. Recrystallization of the C_12_N_2_-H_2_W_12_ precipitate from water or organic solvents was not successful. In “Route 2” (Scheme 1b), using diluted solutions of H_2_W_12_ and C_12_N_2_, only a small amount of precipitate was obtained, and colorless plate crystals were grown from the resulting clear filtrate. Although some single crystals were subjected to X-ray diffraction measurements for structural analysis, only the positions of the H_2_W_12_ anion and part of the C_12_N_2_ cation could be determined (see below). The whole molecular and crystal structures of C_12_N_2_-H_2_W_12_ were revealed using single crystals obtained via “Route 3” (Scheme 1c), which utilized decatungstoeuropate anions ([EuW_10_O_36_]^9−^, EuW_10_) [39]. Sodium salt of the EuW_10_ anion (Na-EuW_10_) was employed as a starting material under hydrothermal conditions, and decomposed during heating to form a mixture of colorless plate crystals of C_12_N_2_-H_2_W_12_.

**Figure 2:**
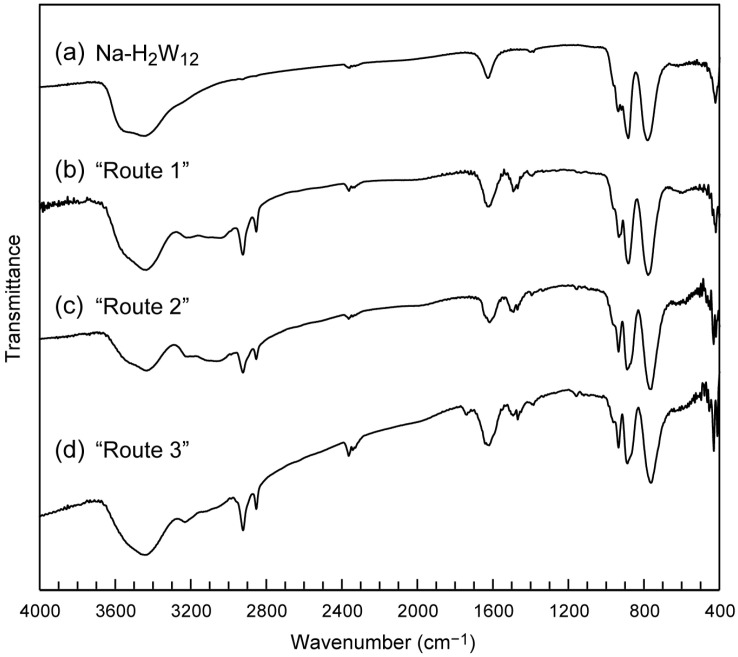
IR spectra of C_12_N_2_-H_2_W_12_ hybrid crystals: (**a**) starting sodium salt of H_2_W_12_ (Na-H_2_W_12_); (**b**) colorless precipitate of C_12_N_2_-H_2_W_12_ obtained using “Route 1”; (**c**) colorless crystals of C_12_N_2_-H_2_W_12_ obtained using “Route 2”; and (**d**) colorless crystals of C_12_N_2_-H_2_W_12_ obtained using “Route 3”.

The products obtained using “Route 1”, “Route 2”, and “Route 3” exhibited almost identical infrared (IR) spectra (Figure 2b–d), showing characteristic peaks of H_2_W_12_ in the range of 400–1000 cm^−1^ (910–980 cm^−1^ [*ν*_as_(W=O_t_)], 840–910 cm^−1^ [*ν*_as_(W-O_b_-W)], and 680–840 cm^−1^ [*ν*_as_(W-O_c_-W)], respectively) [40]. The peaks of C_12_N_2_ (2800–3000 cm^−1^ [*ν*_as_(-CH_2_-)]) were simultaneously observed [41], demonstrating the successful hybridization of the H_2_W_12_ anion and the C_12_N_2_ cation.

**Figure 3:**
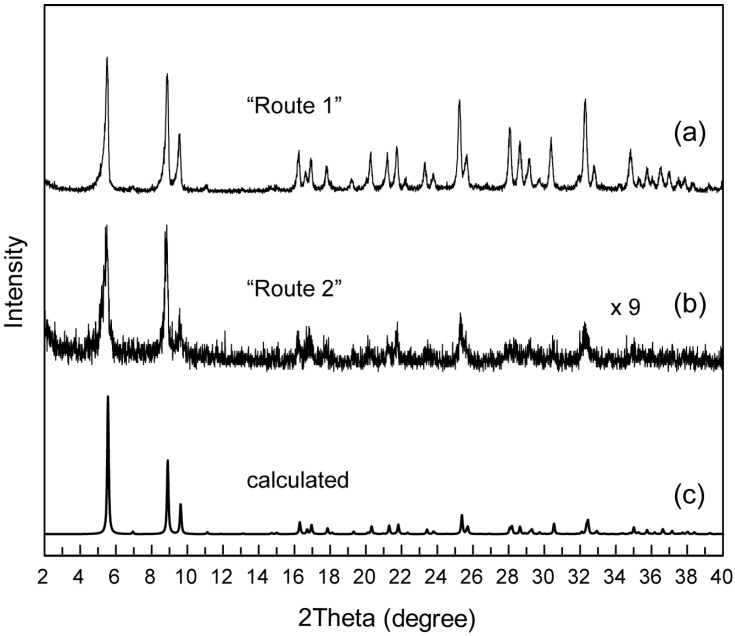
Powder XRD patterns of C_12_N_2_-H_2_W_12_ hybrid crystals: (**a**) colorless precipitate of C_12_N_2_-H_2_W_12_ obtained using “Route 1”; (**b**) colorless crystals of C_12_N_2_-H_2_W_12_ obtained using “Route 2”; and (**c**) calculated pattern from the crystal structure of C_12_N_2_-H_2_W_12_ obtained using “Route 3”.

Powder X-ray diffraction (XRD) patterns obtained using “Route 1” and “Route 2” (Figure 3a,b) were similar to that calculated from the crystal structure of C_12_N_2_-H_2_W_12_ obtained using “Route 3” (Figure 3c). This indicates that the C_12_N_2_-H_2_W_12_ hybrid crystals from “Route 1” and “Route 2” were obtained as pure phases having the same molecular and crystal structures as those of C_12_N_2_-H_2_W_12_ obtained using “Route 3” (see below).

### 2.2. Crystal Strucure of C_12_N_2_-H_2_W_12_ Hybrid Crystals

Colorless plate crystals of C_12_N_2_-H_2_W_12_ suitable for single crystal X-ray diffraction were obtained using “Route 3” as a mixture with brown precipitate. The chemical formula was determined as [H_3_N(CH_2_)_12_NH_3_]_3_[H_2_W_12_O_40_]·4H_2_O together with CHN elemental and IR spectroscopic analyses (Table 1). Three C_12_N_2_ cations (2+ charge) and one Keggin-type H_2_W_12_ anion (6− charge) were associated to neutralize the net charge of the compound. Water molecules of crystallization were also comprised in the crystal lattice of C_12_N_2_-H_2_W_12_; however, Eu^3+^ contained in the starting material was not included (see Section 3).

**Figure 4:**
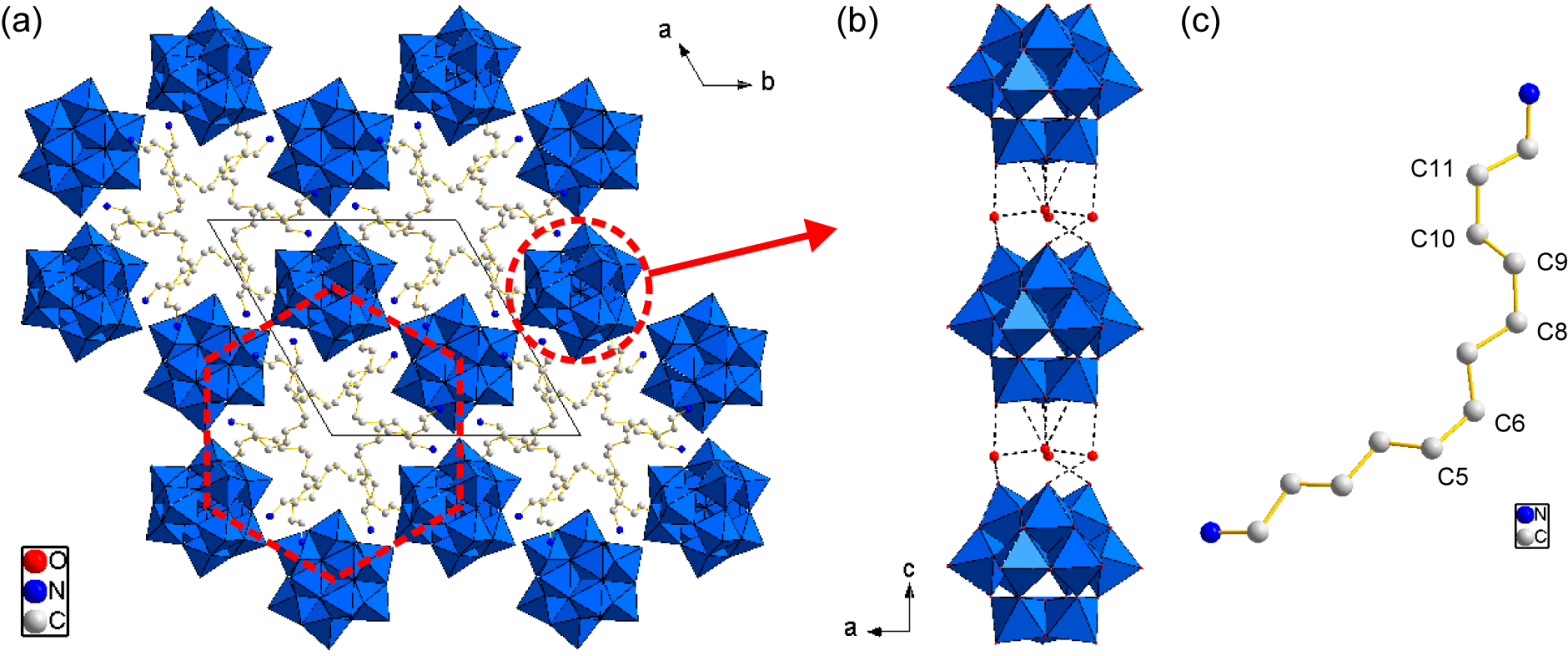
Crystal structure of C_12_N_2_-H_2_W_12_ hybrid crystal obtained using “Route 3” (C: gray, N: blue, O: red; and H_2_W_12_ anions depicted by polyhedral representation). H atoms were omitted for clarity. (**a**) Packing diagram projected along the *c*-axis. A honeycomb-like arrangement of H_2_W_12_ anions is highlighted by a red broken hexagon; (**b**) molecular arrangement of H_2_W_12_ anions and water molecules of crystallization (1D H_2_W_12_-H_2_O chain) viewed along the *b*-axis, with O⋯O short contacts represented by black broken lines; and (**c**) molecular conformation of C_12_N_2_ cation. C atoms relevant to gauche conformation are numbered.

The crystal C_12_N_2_-H_2_W_12_ exhibited a distinct honeycomb-like structure along the *c*-axis (Figure 4a). In this honeycomb-like arrangement, the H_2_W_12_ anions formed a one-dimensional chain structure with water molecules of crystallization (1D H_2_W_12_-H_2_O chains, Figure 4b) via O-H⋯O hydrogen bonds [42]. The O⋯O distances were 2.79–3.18 Å (mean value: 2.99 Å) between the H_2_W_12_ anion and water molecule, and 3.05 Å between water molecules. These 1D H_2_W_12_-H_2_O chains extended along the *c*-axis, separated by the complicatedly bent C_12_N_2_ cations (Figure 4c), resulting in the honeycomb-like arrangement along the *c*-axis. The honeycomb-like arrangement of the H_2_W_12_ anions was confirmed in the crystals obtained using “Route 2” (Figure S1), while only the positions of H_2_W_12_ anions and some carbon atoms were determined. The honeycomb-like arrangement formed from separated 1D molecular chains was similar to another system of POM-surfactant hybrid crystals [43].

As shown in Figure 4c, the bent C_12_N_2_ cation held three C-C bonds with gauche conformation (C5–C6, C8–C9, and C10–C11), which caused the segregation of hydrophilic and hydrophobic sections in the C_12_N_2_-H_2_W_12_ crystal to form a honeycomb-like arrangement of 1D H_2_W_12_-H_2_O chains (Figure 4a,b). The hydrophilic heads of C_12_N_2_ interacted with the H_2_W_12_ anions via N-H⋯O hydrogen bonds [42] (N⋯O distance of 2.79–3.01 Å; mean value: 2.85 Å). Some methylene groups of the C_12_N_2_ cation formed C-H⋯O hydrogen bonds [42] (C⋯O distance of 3.37–3.72 Å; mean value: 3.58 Å) with the H_2_W_12_ anions.

### 2.3. Conductive Property of C_12_N_2_-H_2_W_12_ Hybrid Crystal

**Figure 5:**
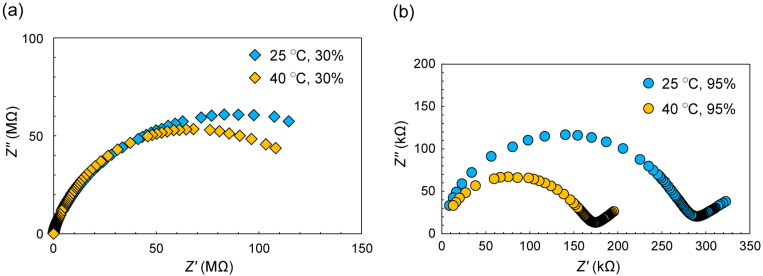
Nyquist spectra of C_12_N_2_-H_2_W_12_ hybrid crystals: (**a**) measured under a low-humidified condition (30% RH) and (**b**) measured under a high-humidified condition (95% RH).

The honeycomb-like structure of the C_12_N_2_-H_2_W_12_ hybrid crystal associated anisotropic 1D alignment of the H_2_W_12_ anions and C_12_N_2_ cations, which may be beneficial to the emergence of conductivity derived from the dissociative protons of C_12_N_2_. The conductivity of the C_12_N_2_-H_2_W_12_ hybrid crystal was evaluated by alternating current (AC) impedance spectroscopy under low- and high-humidified conditions. Figure 5a shows that Nyquist spectra under low humidified conditions (30% RH) exhibited high values in the real part of complex impedance. The conductivities were estimated from the interception values in the horizontal axis of extrapolated semicircular Nyquist spectra to be 1.1 × 10^−8^ S cm^−1^ at 25 °C and 1.3 × 10^−8^ S cm^−1^ at 40 °C. The conductivity values under the high-humidified condition (95% RH) significantly increased to 5.9 × 10^−6^ S cm^−1^ at 25 °C and 9.4 × 10^−6^ S cm^−1^ at 40 °C (Figure 5b). The conductivity values were not high enough; however, they increased by two orders of magnitude from the low-humidified condition to the high-humidified condition, which implied that the C_12_N_2_-H_2_W_12_ hybrid crystal was a moderate proton conductor.

## 3. Discussion

As shown in Scheme 1, C_12_N_2_-H_2_W_12_ hybrid crystals were prepared using three different routes, which essentially employed an ion-exchange reaction. The synthesized products of C_12_N_2_-H_2_W_12_ were the same in their molecular and crystal structures. The C_12_N_2_-H_2_W_12_ hybrid crystals obtained using “Route 1” and “Route 2” were obtained as single phases, whereas the C_12_N_2_-H_2_W_12_ obtained using “Route 3” was a mixture. This was possibly due to the higher synthetic temperature in “Route 3” (150~200 °C), which caused the emergence of another phase as brown precipitate (Figure S2).

The difference between “Route 1” and “Route 2” was the concentration of the starting H_2_W_12_ and C_12_N_2_ solutions. In “Route 1”, concentrated solutions (2.0 × 10^−2^ mol/L for H_2_W_12_ and 4.9 × 10^−2^ mol/L for C_12_N_2_) were applied to obtain crystalline precipitate of C_12_N_2_-H_2_W_12_ at a high yield (~90%). The C_12_N_2_-H_2_W_12_ precipitate from “Route 1” was highly crystalline, and unit cell parameters (*a* = 18.459(4), *b* = 18.459(4), *c* = 12.696(4) Å, α = 90.000, *β* = 90.000, *γ* = 120.000°, and *V* = 3746.4(16) Å^3^) were found using the powder XRD pattern (Figure 3a). These values were similar to those revealed by single crystal structure analyses (Table 1), indicating that the C_12_N_2_-H_2_W_12_ precipitate from “Route 1” and single crystals from “Route 2” (Figure S1) and “Route 3” (Figure 4) had the same crystal structures. Once prepared, the C_12_N_2_-H_2_W_12_ precipitate could not be recrystallized from water or organic solvents, probably due to low solubility.

On the other hand, “Route 2”, utilizing diluted solutions (5.0 × 10^−4^ mol/L for H_2_W_12_ and 1.5 × 10^−3^ mol/L for C_12_N_2_), yielded a small amount of precipitate just after the H_2_W_12_ and C_12_N_2_ solutions were combined; this enabled the growth of C_12_N_2_-H_2_W_12_ crystals from the resulting filtrate. Although the crystals obtained using “Route 2” were not quite suitable for the single crystal X-ray diffraction, the honeycomb-like arrangement of H_2_W_12_ was confirmed, as shown in Figure S1.

“Route 3” was carried out under hydrothermal conditions to result in the formation of single crystals of C_12_N_2_-H_2_W_12_. A synthetic temperature higher (150~200 °C) than “Route 1” and “Route 2” (50 °C) seemed effective for crystal growth of C_12_N_2_-H_2_W_12_ crystals having relatively low solubility (<~5.0 × 10^−4^ mol/L). The starting material, Na-EuW_10_, decomposed under hydrothermal conditions to form single crystals of C_12_N_2_-H_2_W_12_ and brown precipitate (Figure S2). The presence of Eu^3+^ was not observed in the crystal structure of C_12_N_2_-H_2_W_12_, but was observed in the brown precipitate that exhibited emission owing to Eu^3+^ (Figure S2c). The emission spectrum and emission decay time (2.3 ms) were similar to those of Na-EuW_10_ [39]. The IR peaks of the brown solid were relatively unclear, but showed some signals observed in Na-EuW_10_ and C_12_N_2_ (Figure S2a). The brown solid did not contain the crystalline C_12_N_2_-H_2_W_12_ (Figure S2b); therefore, it may have been a hybrid material of the remaining EuW_10_ anion and C_12_N_2_ cation.

The crystal packing of C_12_N_2_-H_2_W_12_ possessed a striking honeycomb-like structure, which is rare [30,43]; long bolaamphiphiles or single-headed surfactants hybridized with POM anions seem to prefer layer structures [18,19,20,21,22,23,24,34,36,37,38]. It is not clear why the C_12_N_2_-H_2_W_12_ hybrid crystal packed in the honeycomb-like structure; however, the formation of 1D H_2_W_12_-H_2_O chains were crucial [43]. The formation of layer structure in POM–surfactant hybrid crystals requires the formation of two-dimensional (2D) layers consisting of chemical components [28,29,34]. In the C_12_N_2_-H_2_W_12_ crystal, hydrogen-bonded 1D H_2_W_12_-H_2_O chains formed, and the molecular interaction between these 1D H_2_W_12_-H_2_O chains may not have been anisotropically strong enough to form 2D alignment, which resulted in more isotropic alignment with a honeycomb-like structure.

The C_12_N_2_-H_2_W_12_ hybrid crystal had dissociative C_12_N_2_ protons with 1D alignment due to the honeycomb-like structure, which may be promising for the emergence of proton conductivity. The conductivities were 10^−8^ S cm^−1^ order under low-humidified conditions and 10^−6^ S cm^−1^ order under high-humidified conditions, which were relatively low values. Adding humidity improved the conductivities by two orders of magnitude to ~10^−6^ S cm^−1^, suggesting the relevance of water molecules in the conduction mechanism. As mentioned above, the C_12_N_2_-H_2_W_12_ hybrid crystal had water molecules of crystallization forming 1D H_2_W_12_-H_2_O chains. These plausibly rigid 1D H_2_W_12_-H_2_O chains may have decreased the mobility of water molecules in the crystal lattice of C_12_N_2_-H_2_W_12_, resulting in low conductivities. The C_12_N_2_-H_2_W_12_ hybrid crystals were stable until 250 °C, as shown in TG profile (Figure S3), and its conductivity may have increased under a higher temperature (>100 °C). Although the conductivity of C_12_N_2_-W_12_ was not high, the relevance between the compound structure and proton conductivity could provide helpful suggestions regarding the construction of effective proton conductors.

## 4. Materials and Methods

### 4.1. General Procedures and Instrumental Methods

Chemical reagents purchased from commercial sources (FUJIFILM Wako Pure Chemical Corporation, Osaka, Japan, and Tokyo Chemical Industry Co., Ltd. (TCI), Tokyo, Japan) were utilized as received. Solid 1,12-dodecamethylenediammonium chloride ([H_3_N(CH_2_)_12_NH_3_]Cl_2_, C_12_N_2_-Cl) was prepared by adding equimolar hydrochloric acid to 1,12-dodecanediamine. Sodium decatungstoeuropate (Na_9_[EuW_10_O_36_]·32H_2_O, Na-EuW_10_) was prepared according to the literature [39].

Infrared (IR) spectra were measured using a Jasco FT/IR-4200ST spectrometer (KBr pellet method). Powder X-ray diffraction (XRD) patterns were recorded using a Rigaku MiniFlex300 diffractometer (Cu Kα radiation, *λ* = 1.54056 Å) under ambient atmosphere. Unit cell parameters were found using DASH [44]. CHN (carbon, hydrogen, and nitrogen) elemental analyses were conducted using a PerkinElmer 2400II elemental analyzer. Thermal gravimetric (TG) analyses were carried out using a Seiko Instruments TG/DTA-6200 at a heating rate of 10 °C min^−1^ under a nitrogen atmosphere. Emission spectra were measured at room temperature using a Jasco FP-8300 fluorescence spectrometer. Alternating current (AC) impedance spectra were recorded using an Agilent technologies 4284A or a HIOKI 3532-50 LCR meter (frequency range: 5 to 5.0 × 10^6^ Hz) coupled with an Espec SH-641 bench-top-type temperature and humidity chamber with pelletized samples sandwiched with Pt electrodes.

### 4.2. Syntheses

#### 4.2.1. Route 1

Sodium metatungstate (Na_6_[H_2_W_12_O_40_]·*n*H_2_O (Na-H_2_W_12_), 0.70 g (0.20 mmol) was dissolved in 10 mL of distilled water, and 1,12-dodecanediamine (0.12 g, 0.59 mmol) was dissolved in 10 mL of distilled water by adding 2 mL of 1 M HCl. These solutions were heated to ca. 50 °C and mixed together at ca. 50 °C. The resulting suspension was maintained at 42 °C for 12 days, and then colorless precipitate was filtered and dried to obtain colorless crystalline precipitate of C_12_N_2_-H_2_W_12_ (0.61 g, yield 90%). This crystalline precipitate of C_12_N_2_-H_2_W_12_ could not be recrystallized from water or organic solvent. Analysis: calculated for C_36_H_100_N_6_W_12_O_40_: C: 12.26, H: 2.86, and N: 2.38%. Found: C: 12.21, H: 2.84, and N: 2.21%. IR (KBr disk): 3223 (w), 3041 (w), 2924 (m), 2853 (m), 1492 (w), 1469 (w), 932 (m), 882 (s), 777 (s), 595 (w), 435 (w), and 419 (w) cm^−1^.

#### 4.2.2. Route 2

Na-H_2_W_12_ (0.036 g, 0.010 mmol) was dissolved in 20 mL of distilled water, and 1,12-dodecanediamine (0.0078 g, 0.038 mmol) was dissolved in 25 mL of ethanol. These solutions were heated to ca. 50 °C, mixed together at ca. 50 °C with stirring for 10 min, and then filtered. The resulting colorless clear filtrate was maintained at 42 °C to form colorless plate crystals of C_12_N_2_-H_2_W_12_. Some crystals were subjected to single crystal X-ray diffraction measurements; IR (KBr disk): 3217 (w), 3063 (w), 2924 (m), 2853 (m), 1491 (w), 1469 (w), 934 (m), 889 (s), 763 (s), 623 (w), 430 (w), and 418 (w) cm^−1^.

#### 4.2.3. Route 3

Na-EuW_10_ (0.50 g, 0.16 mmol) and C_12_N_2_-Cl (0.280 g, 1.0 mmol) were dissolved in 10 mL of distilled water. A more diluted condition (Na-EuW_10_: 0.22 g, 0.070 mmol; C_12_N_2_-Cl: 0.064 g, 0.24 mmol) was also used. The Na-EuW_10_ solution was poured into 30 mL Teflon beaker, and then the solution of C_12_N_2_-Cl was added. The Teflon beaker was transferred into a stainless container, heated at 150~200 °C for 96 h, and then cooled to room temperature for 48 h. The resulting product, containing colorless plate crystals of C_12_N_2_-H_2_W_12_ and brown precipitate, was filtered and dried. The crystals were suitable for single crystal X-ray diffraction measurements.

### 4.3. Crystal Structure Determination

X-ray diffraction data for the C_12_N_2_-H_2_W_12_ crystals were collected using a Rigaku R-AXIS RAPID diffractometer with PROCESS-AUTO [45] or an XtaLAB P200 diffractometer with CrysAlisPro [46]. Both instruments used graphite monochromated Mo Kα radiation. Crystal structures were solved using SHELXS (Version 2013/1) [47] or SHELXT (Version 2018/2) [48], and refined using the full-matrix least-squares using SHELXL (Version 2018/3) [47].

## Figures and Tables

**Table 1:** Crystallographic data.

Compound	C_12_N_2_-H_2_W_12_
Chemical formula	C_36_H_92_N_6_W_12_O_44_
Formula weight	3519.35
Crystal system	trigonal
Space group	*P*3 (No. 147)
*a* (Å)	18.3644 (4)
*b* (Å)	18.3644 (4)
*c* (Å)	12.7069 (3)
*α* (°)	90.000
*β* (°)	90.000
*γ* (°)	120.000
*V* (Å^3^)	3711.28 (17)
*Z*	2
*ρ*_calcd_ (g cm^−3^)	3.149
*T* (K)	296 (2)
Wavelength (Å)	0.71075
*μ* (mm^−1^)	18.604
No. of reflections measured	60,648
No. of independent reflections	5667
*R*_int_	0.0619
No. of parameters	301
*R*_1_ (*I* > 2*σ*(*I*))	0.0227
*wR*_2_ (all data)	0.0501

## Data Availability

Further details of the crystal structure investigation (CCDC 2232675) can be obtained free of charge via www.ccdc.cam.ac.uk/data_request/cif (accessed on 24 December 2022), or by emailing data_request@ccdc.cam.ac.uk, or by contacting The Cambridge Crystallographic Data Centre, 12 Union Road, Cambridge CB2 1EZ, UK; Fax: +44-1223-336033.

## Supplementary Materials

The following supporting information can be downloaded at: https://www.mdpi.com/article/10.3390/ijms24032824/s1.
