# Supplementary material for: An Inorganic–Organic Hybrid Framework Composed of Polyoxotungstate and Long-Chained Bolaamphiphile
Source: Int J Mol Sci. 2023 Feb 1;24(3):2824. doi: 10.3390/ijms24032824 (PMC9917333; doi:10.3390/ijms24032824)
Supplement: Supplementary file 1 [file ijms-24-02824-s001.zip › ijms-2152788-supplementary.pdf]

Supporting Information

An Inorganic-Organic Hybrid Framework Composed of Polyoxotungstate and Long-Chained Bolaamphiphile

Haruka Ikuma [1], Shunsuke Aoki [1], Kai Kawahara [1], Seiji Ono [2], Hironori Iwamatsu [2], Jun Kobayashi [1], Yoshiki Kiyota [1], Yosuke Okamura [2], Masashi Higuchi [2] and Takeru Ito [1,*]

[1] Department of Chemistry, School of Science, Tokai University, Kanagawa 259-1292, Japan

[2] Department of Applied Chemistry, School of Engineering, Tokai University, Hiratsuka, Kanagawa 259-1292, Japan

*Correspondence: takeito@keyaki.cc.u-tokai.ac.jp

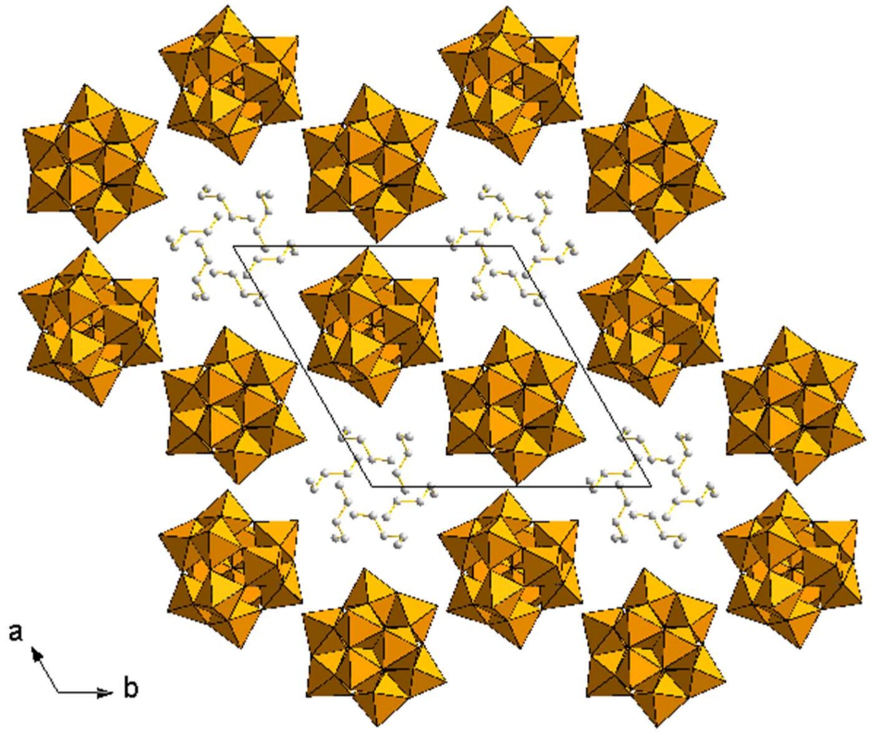

Figure S1. Crystal structure of $C_{12}N_2$-H_2W_{12} hybrid crystal obtained by "Route 2". (C: gray; H_2W_{12} anions are depicted by polyhedral representation). Only the position of H_2W_{12} anions and some carbon atoms were determined. Crystallographic data: $P\bar{3}$ (#147), a = 18.2778(3), b = 18.2778(3), c = 12.7263(3) Å, α = 90.000, β = 90.000, γ = 120.000 °, V = 3681.97(12) Å^3, R_1 = 0.2763, wR_2 = 0.6080.

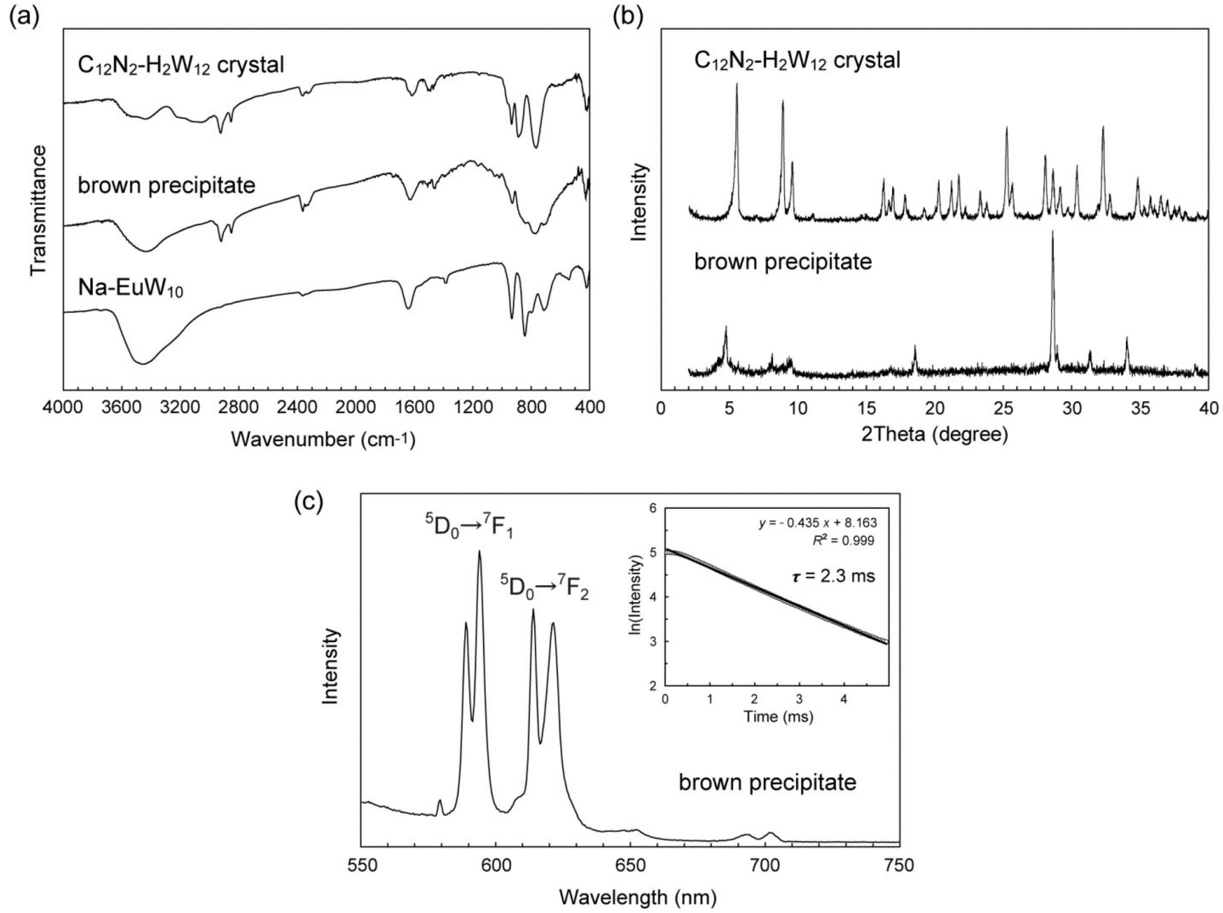

Figure S2. Structural information of brown precipitate as by-product obtained by "Route 3": (**a**) IR spectra; (b) Powder XRD patterns; (**c**) Emission spectrum excited by irradiation with a wavelength of 254 nm. Some assignments of the emission peaks are represented. Inset: emission decay monitored on 594 nm emission excited by 254 nm irradiation.

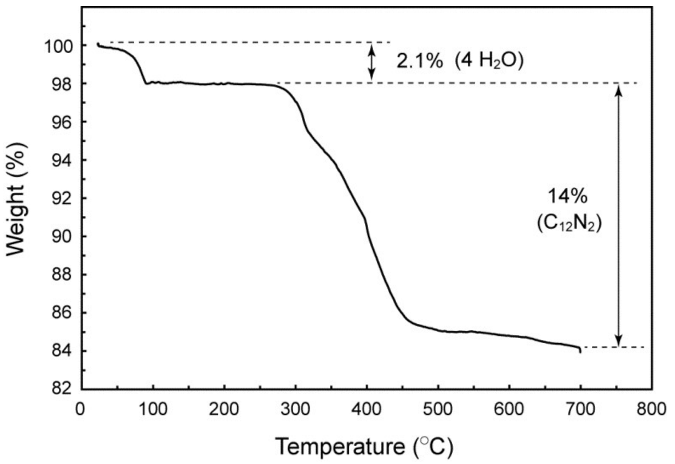

Figure S3. TG profile of $C_{12}N_2$-H_2W_{12} hybrid crystal.
